# Major adverse cardiovascular event definitions used in observational analysis of administrative databases: a systematic review

**DOI:** 10.1186/s12874-021-01440-5

**Published:** 2021-11-06

**Authors:** Elliott Bosco, Leon Hsueh, Kevin W. McConeghy, Stefan Gravenstein, Elie Saade

**Affiliations:** 1 Department of Health Services, Policy, and Practice, Brown University School of Public Health, 121 South Main Street, Box G-S121-3, Providence, RI 02912 USA; 2 Center for Gerontology and Healthcare Research, Brown University School of Public Health, RI Providence, USA; 3 Department of Medicine, Warren Alpert Medical School of Brown University, Providence, RI USA; 4 Center of Innovation in Long-Term Services and Supports, Providence Veterans Affairs Medical Center, Providence, RI USA; 5 Division of Infectious Diseases and HIV Medicine, University Hospitals Cleveland Medical Center, Cleveland, OH USA; 6 School of Medicine, Case Western Reserve University, Cleveland, OH USA

**Keywords:** Observational study, Reproducibility, Acute myocardial infarction, Stroke, Heart failure, Acute coronary syndrome, Cardiovascular disease

## Abstract

**Background:**

Major adverse cardiovascular events (MACE) are increasingly used as composite outcomes in randomized controlled trials (RCTs) and observational studies. However, it is unclear how observational studies most commonly define MACE in the literature when using administrative data.

**Methods:**

We identified peer-reviewed articles published in MEDLINE and EMBASE between January 1, 2010 to October 9, 2020. Studies utilizing administrative data to assess the MACE composite outcome using International Classification of Diseases 9th or 10th Revision diagnosis codes were included. Reviews, abstracts, and studies not providing outcome code definitions were excluded. Data extracted included data source, timeframe, MACE components, code definitions, code positions, and outcome validation.

**Results:**

A total of 920 articles were screened, 412 were retained for full-text review, and 58 were included. Only 8.6% (*n* = 5/58) matched the traditional three-point MACE RCT definition of acute myocardial infarction (AMI), stroke, or cardiovascular death. None matched four-point (+unstable angina) or five-point MACE (+unstable angina and heart failure). The most common MACE components were: AMI and stroke, 15.5% (*n* = 9/58); AMI, stroke, and all-cause death, 13.8% (*n* = 8/58); and AMI, stroke and cardiovascular death 8.6% (*n* = 5/58). Further, 67% (*n* = 39/58) did not validate outcomes or cite validation studies. Additionally, 70.7% (*n* = 41/58) did not report code positions of endpoints, 20.7% (*n* = 12/58) used the primary position, and 8.6% (*n* = 5/58) used any position.

**Conclusions:**

Components of MACE endpoints and diagnostic codes used varied widely across observational studies. Variability in the MACE definitions used and information reported across observational studies prohibit the comparison, replication, and aggregation of findings. Studies should transparently report the administrative codes used and code positions, as well as utilize validated outcome definitions when possible.

**Supplementary Information:**

The online version contains supplementary material available at 10.1186/s12874-021-01440-5.

## Background

Cardiovascular disease is the leading cause of death in the United States, making it a common target for interventional research [1, 2]. Due to this, the composite endpoint of “major adverse cardiovascular events” (MACE) is an increasingly common primary outcome of interest. In 2008, the United States Food and Drug Administration (FDA), followed by the European Medicines Agency (EMA) in 2012, provided guidance on utilizing a three-point MACE outcome, which includes acute myocardial infarction (AMI), stroke, and cardiovascular mortality in all trials evaluating the cardiovascular safety of diabetic agents [3]. Some trials have utilized a four-point MACE as well, by including hospitalization for unstable angina or revascularization procedures [3, 4]. Five-point MACE further expands on this with the inclusion of heart failure (HF). While MACE is now a better-defined and more ubiquitous outcome among RCTs, its use in observational studies to assess the safety and real-world effectiveness of therapies remains less clear.

Observational studies using large administrative databases can evaluate population-level use and outcomes of therapies in an efficient and cost-effective way [5]. However, several common issues with observational studies limit their reproducibility and comparability to RCTs, thus limiting the utility of MACE as a composite outcome. First, in comparison to RCTs, observational studies often do not consistently report their protocols, including outcome definitions [6]. To allow for useful comparisons between observational studies and RCTs, improved standardization and transparency is needed [7, 8]. Even when MACE components are well-defined, such as AMI or stroke, another challenge encountered in observational studies is how to define outcomes using diagnosis codes available from administrative data. For example, the *International Classification of Diseases* (ICD) is a commonly used coding system for medical reimbursement and is one of the most frequent sources of information available in administrative databases [9]. Unfortunately, ICD codes can be prone to errors and diagnosis misclassifications, as they are primarily collected for reimbursement purposes and rely on clinical documentation that can vary across settings and providers [5]. Though several studies have attempted to validate the diagnosis codes for commonly used MACE components (e.g., AMI, stroke, and HF), with positive predictive values of upwards of 80–90%, it is unclear how these codes have been taken up in MACE composite outcome definitions [10–12]. For these reasons, the current use of MACE in observational studies warrants further investigation.

Thus, the purpose of this review was to systematically determine the most common definitions of MACE employed in observational studies using administrative data. With that, our objectives were: i) assess each study’s definition of MACE components (e.g., AMI, stroke), ii) assess the diagnostic criteria used for outcome ascertainment such as codes used and position of codes, and iii) assess whether outcomes had been validated. We hypothesized that, across observational studies, there is great variability in the definitions of MACE used and minimal alignment with the classic three, four, or five-point MACE outcomes. Our hope is that this work will promote a standard approach to the definition of MACE in future studies, allowing for the improved transparency and reproducibility of observational studies.

## Methods

### Search strategy and selection criteria

The protocol for this systematic review is based on the 2015 Preferred Reporting Items for Systematic Reviews and Meta-Analysis Protocols (PRISMA-P) statement [13]. The protocol was not registered in the International Prospective Register of Systematic Reviews (PROSPERO), but the original protocol and modifications are included in Supplementary Text 1 (see Additional file 1). The reporting of this review is based on the 2020 PRISMA statement and the checklist is provided in Supplementary Table 1 (see Additional file 2) [14]. We searched MEDLINE and EMBASE for literature published from January 1, 2010 to October 9, 2020 that defined composite MACE as a primary or secondary outcome in studies utilizing administrative databases. A 10-year lookback was used in order to restrict to the most recently published studies. The search strategy was developed by the authors using the search terms presented in Supplementary Text 2 (see Additional file 3) and was performed on October 9, 2020. A standardized protocol for study abstract screening and full-text review was developed and piloted using the same 20 studies among the reviewers (EB, ES, LH), and is further described in Supplementary Text 1 (see Additional file 1). The abstracts from each record were initially screened for relevance by three independent reviewers (EB, ES, LH) using Abstrackr (http://abstrackr.cebm.brown.edu/), a free semi-automated abstract screening tool [15]. Afterwards, we performed a full-length text review of the abstracts that were retained after screening (EB, ES, LH).

We included studies that identified MACE composite endpoints as the primary or secondary study outcome, used administrative data sources in the study, and defined MACE using *International Classification of Diseases, Clinical Modification, Ninth Revision* (ICD-9-CM) or *Tenth Revision* (ICD-10-CM) diagnosis codes. The included studies did not need to overtly name “major adverse cardiovascular events” as the primary or secondary outcome to be included into this study, as many definitions and terms are used across studies. For example, MACE may be referred to as “adverse” or “acute cardiovascular events”, “major adverse cardiovascular and cerebrovascular events” or simply “cardiovascular events”, among other terms. Thus, we included studies if the composite outcome that was studied utilized a combination of multiple MACE endpoints. We excluded reviews, meta-analyses, conference abstracts, editorials, papers whose primary or secondary outcome was not a composite of multiple MACE endpoints, and those that did not use or report ICD-9-CM or ICD-10-CM codes to define MACE endpoints. Any uncertainty with regards to inclusion or exclusion of an individual paper was resolved by consensus of the three reviewers.

### Data extraction and synthesis

After each of the independent reviewers determined that a full-text met inclusion criteria for the study, definitions for composite MACE outcomes were extracted into a shared document using a standardized protocol by two reviewers (LH, EB). ICD-9-CM and ICD-10-CM codes were also extracted for each MACE endpoint, and were acquired either through full-length text review or through review of published supplements. The position of the diagnosis code required in the study outcome criteria was recorded as primary position, any position, or not reported. Further, information on whether the study outcomes had been previously validated was assessed in each individual study or review of supplements, and citations were extracted. Each study’s administrative data source and study years were also recorded.

Once data were extracted from included studies, we categorized the specific definitions of MACE based on the individual components referred to by the authors as opposed to the specific diagnosis codes used. We made this decision due to the lack of consistency in diagnosis codes used across studies and chose to categorize based on the MACE components that the authors reported. Component definitions of MACE included: AMI, acute coronary syndrome or ischemic heart disease (ACS/IHD), stroke (either ischemic or hemorrhagic stroke), revascularization procedures, cardiovascular (CV) death, and all-cause death. Of note, due to the variability of outcomes used across studies, the ACS/IHD component reflects the following definitions used by study authors: ACS, IHD, coronary artery disease (CAD), and unstable angina (UA). Overall, we performed a qualitative assessment of the evidence only, and did not perform a meta-analysis or strength of evidence assessment due to the nature of the research questions.

## Results

### Included studies

**Fig. 1:**
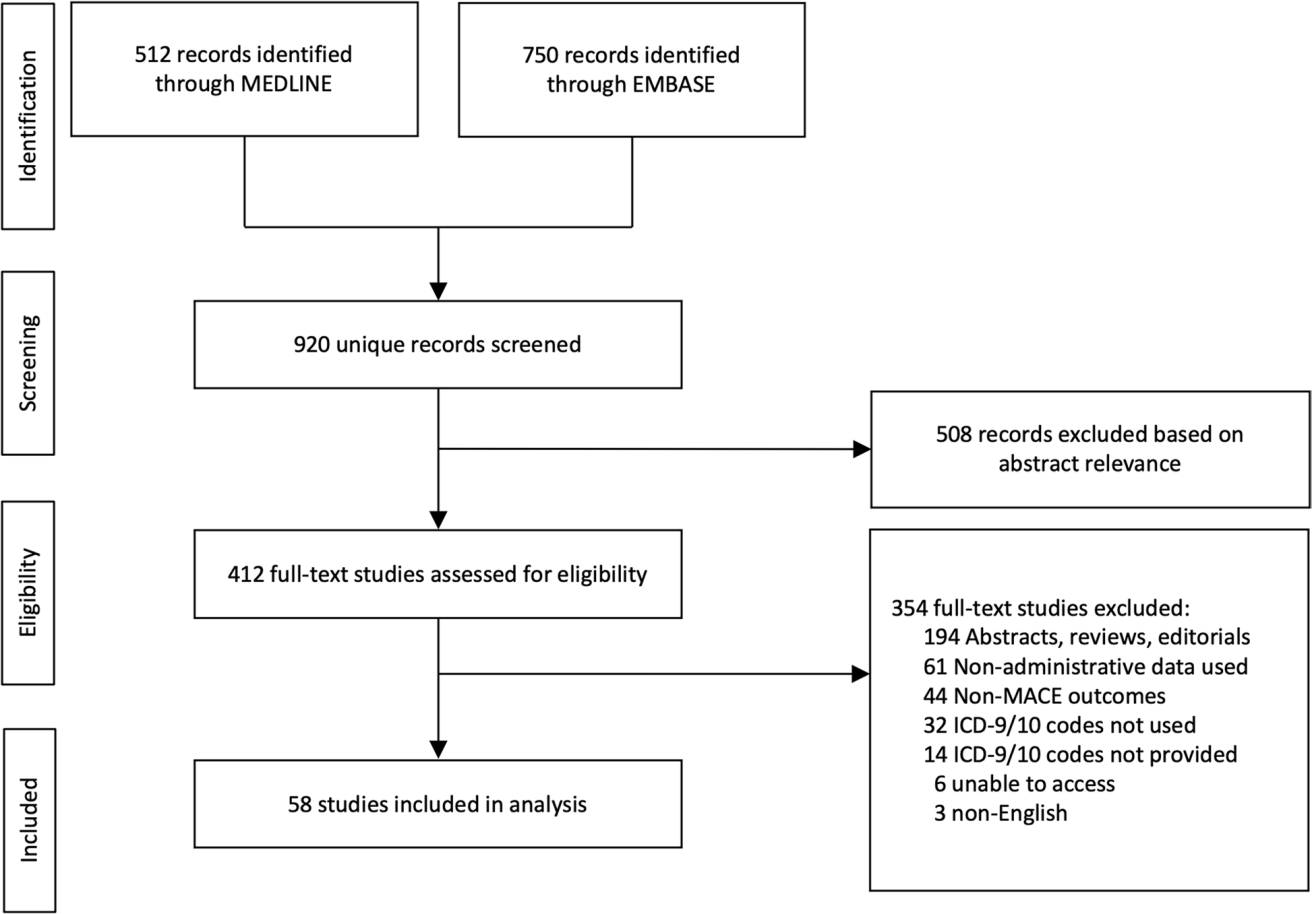
PRISMA flow diagram for included studies. Abbreviations: MACE, major adverse cardiovascular events; ICD, International Classification of Diseases

Our search of MEDLINE and EMBASE yielded 920 unique articles, 412 of which were retained for full-text review after abstract screening (Fig. 1). After excluding 354 studies during full-text review, 58 studies were included in the final analysis.

### Overall studies

The included observational studies utilized a range of outcome components to define MACE (Table 1). The majority of the included studies utilized ICD-9-CM to define outcomes. There was poor concordance of MACE definitions used in the included studies when compared to the three-point, four-point, and five-point definitions of MACE commonly used in randomized controlled trials (RCTs). For instance, 8.6% (5/58) of observational studies used a MACE definition that matched the three-point definition of MACE (AMI, stroke, CV death) while no studies matched the four-point or five-point definitions (Table 2). Across all included studies, the most common MACE component definitions were: AMI, stroke, 15.5% (9/58); AMI, stroke, all-cause death, 13.8% (8/58); and AMI, stroke, CV death 8.6% (5/58) (Table 3). Overall, 67% of studies (39/58) did not perform a validation or provided no citations validating the outcome definitions used. Additionally, 70.7% (41/58) of studies did not report the position of diagnosis codes used in endpoints, while 20.7% (12/58) used the primary diagnosis position and 8.6% (5/58) used any position.

**Table 1:** MACE definitions based on publication

Study Citation and Publication Year	Data Source and Time Period	MACE Definition	ICD-9/10 Codes Diagnosis or Procedure Position	Validation
Chapman et al. (2010) [16]	IMS LifeLink: US Health Plan Claims database from 2004 to 2005	AMI, other non-AMI IHD, HF, stroke/TIA, PVD, cardiac revascularization procedure, carotid endarterectomy	AMI: 410.xx, 412; other non-AMI IHD: 411.xx, 413.xx, 414.xx, 427.xx, V45.81, V45.82; stroke/TIA: 433.xx, 434.xx, 435.x, 436, 437.0, 437.1, 438; PVD: 440, 440.1, 443.xx (ICD-9). (Authors did not cite ICD codes for carotid endarterectomy or other revascularization procedures.) Primary position.	NR
Gudbjornsson et al. (2010) [17]	Icelandic Medicines Registry from 2001 to 2003	AMI, UA, stroke	AMI: I21; UA: I20.0; stroke: I63 (ICD-10). NR	NR
Charlot et al. (2011) [18]	Danish national patient registry from 1997 to 2006	AMI, stroke, CV death	AMI: I21, I22; Stroke: I60–I69, G45 (ICD-10). (No specific codes were listed for CV death). Any position.	Validated through data linkage between the Danish national patient registry and patient level data collected through the Danish MONICA registry [19] and the Copenhagen City Heart Study [20]
Kociol et al. (2011) [21]	Medicare claims linked to the ADHERE-Core registry from 2001 to 2004	AMI, stroke	AMI: 410.× 1; stroke: 433.x, 434.x (ICD-9). Primary position.	NR
Chen et al. (2012) [22]	Taiwan National Health Research Insurance database from 2000	AMI, ischemic stroke	AMI: 410.xx; ischemic stroke 433.xx, 434.xx, 436, 437.1 (ICD-9). NR	Authors cite a study validating incident events utilizing these specific ICD codes when compared with direct patient chart review [23]
Degli Esposti et al. (2012) [24]	Multiple databases of the Local Health Unit of Florence from 2004 to 2007	AMI, stroke, all cause death	AMI: 410.x; stroke: 430–438.x (ICD-9). NR	NR
Parker et al. (2012) [25]	Cardiovascular Research Network Hypertension Registry from 2000 to 2009	IHD, stroke, PVD, HF	IHD: 410.xx–414.xx; stroke: 430.xx–434.xx, 436.xx, 852.0, 852.2. 852.4, 853.0; PVD: 441.3–441.7, 443.9, 444.0, 444.2; HF: 428.xx, 402.xx, 398.91 (ICD-9). Primary position.	NR
Karthikesalingam et al. (2013) [26]	Hospital Episode Statistics data from 2006 to 2011	AMI, stroke, aortoiliac or peripheral arterial thrombosis or embolism, emergency amputation, open/endovascular lower limb revascularization	AMI: I21, I22; stroke: I63, I64, I69.4, I69.8 (ICD-10). NR	NR
Mortensen et al. (2014) [27]	VA Health Care System administrative and clinical databases from 2001 to 2012	AMI, HF, cardiac arrhythmia	AMI: 410, 410.0–410.9, 411, 411.1, 412, 414.2, 414.8, 429.71, 429.79; HF: 402, 402.1, 402.10, 402.11, 402.9, 402.90, 402.91, 404, 414.19, 425.4, 428, 428.0, 428.1, 428.2, 428.20–428.23, 428.3, 428.30–428.33, 428.4, 428.40–428.43, 428.9, 429.1, 429.4, 997.1; cardiac arrhythmia: 427, 427.0, 427.1–427.5, 427.31, 427.32, 427.41, 427.42, 427.8, 427.81, 427.89, 427.9, 429.4, 997.1, V12.53 (ICD-9). NR	NR
Neumann et al. (2014) [28]	Système National d’Information Inter-Régimes de l’Assurance Maladie from 2008 to 2011	IHD, ischemic stroke	IHD: I21–24; ischemic stroke: I63, I65, I66. (ICD-10). Any position.	NR
Vanasse et al. (2014) [29]	Régie de l’assurance maladie du Québec from 2006 to 2007	CV disease, CV death	CV disease: 410–414, 428, 430–438 (ICD-9). I20–I25, I50, I60-I69 (ICD-10). Any position.	NR
Chou et al. (2015) [30]	National Health Insurance Research Database of Taiwan from 2001 to 2010	AMI, stroke, HF, cardiac arrhythmia, cardiac shock, cardiac revascularization procedure	AMI: 410–410.9; stroke: 430–437; HF: 428.0–428.10, cardiac arrhythmia: 26.0, 426.12–426.13, 426.51, 426.52, 426.54, 427.1, 427.4, 427.41, 427.42, 427.5; cardiac shock: 785.51; revascularization: 36.0–36.03, 36.05–36.09, 36.1–36.99, V45.8, 36.0–36.9 (ICD-9). NR	NR
Desai et al. (2015) [31]	SUPREME-DM consortium database from 2005 to 2011	ACS, stroke, HF	ACS: 410.0–410.9, 411.1–411.8; stroke: 430–432.9, 433–434.9; HF: 428–428.9 (ICD-9). Primary position.	Authors cited multiple studies that have shown good PPVs using these specific ICD codes [32–34]
Doll et al. (2015) [35]	Medicare claims linked to the ACTION Registry-GWTG registry from 2007 to 2010	AMI, stroke, all cause death, cardiac revascularization procedure	AMI: 410.× 1; stroke: 430.x, 431.x, 432.x, 433.× 1, 434.× 1, 436.x, 437.1, 437.9, 997.02; revascularization: 00.66, 36.0, 36.1, 36.2, 36.3 (ICD-9). Primary position.	NR
Jernberg et al. (2015) [36]	Swedish National Inpatient Register, Swedish Prescribed Drug Register, and Swedish Cause of Death Register from 2006 to 2011	AMI, stroke, CV death	AMI: I21; stroke: I61-I64; CV death: I00-I99 (ICD-10). NR	Authors cited a study which reported that > 95% of MI diagnoses were valid in the National Inpatient register [37]. No validation was reported for stroke or CV death.
Korsnes et al. (2015) [38]	MarketScan Commercial Claims and Encounters and Medicare Supplemental Database from 2006 to 2011	AMI, ischemic stroke	AMI: 410.xx; ischemic stroke: 433.xx, 434.xx, 436.xx (ICD-9). Primary position.	NR
Roussel et al. (2015) [39]	French National Health Insurance Information System from 2008 to 2010	AMI, ischemic stroke, all cause death, above the ankle amputations	AMI: I21-I24; ischemic stroke: I63, I65, I66 (ICD-10). (Authors did not use ICD codes for above the ankle amputations.) NR	NR
Sirois et al. (2015) [40]	Quebec health insurance board databases, Quebec registry of hospitalization databases from 1998 to 2004	AMI, strokes	AMI: 410; stroke: 430–431, 433.× 1, 434.× 1 and 435 (ICD-9). Any position	Authors cite a study that validated the accuracy of coding hospital discharge data of AMI and found a PPV of 96% utilizing this specific ICD code for AMI [41]. No validation was reported for stroke.
Stochkendahl et al. (2015) [42]	Danish National Patient Register from 2006 to 2011	ACS, stroke, CV death, cardiac revascularization procedure	ACS: I21-I23, I24.0, I24.8, I24.9; stroke: I60-I69; CV death: I00-I99 (ICD-10). (Authors did not cite ICD codes for cardiac revascularization procedures.) NR	NR
Blin et al. (2016) [43]	Echantillon Généraliste des Bénéficiaires database from 2007 to 2010	AMI, stroke/TIA, all cause death	AMI: I21.0-I21.9, I22.0–22.9; stroke/TIA: I60-I64, G45 (ICD-10). Primary position.	NR
Cheng et al. (2016) [44]	Taiwan National Health Research Insurance database from 2004 to 2015	AMI, ischemic stroke, hemorrhagic stroke, all cause death	AMI: 410; ischemic stroke: 434–437; hemorrhagic stroke: 430–432 (ICD-9). NR	NR
Chuang et al. (2016) [45]	Taiwan National Health Research Insurance database from 1996 to 2011	ACS, ischemic stroke, hemorrhagic stroke	ACS: 410, 411; ischemic stroke: 433–438; hemorrhagic stroke: 430–432 (ICD-9). NR	NR
Fortuna et al. (2016) [46]	HealthPartners Medical Group electronic medical record (EPIC) from 2007 to 2009	AMI. stroke, PCI	AMI: 410.xx; stroke: 430.xx-434.xx, 436.xx, 852.0, 852.2, 852.4, 853.0 (ICD-9); PCI: 92980–92,996 (CPT). NR	Authors cited a study validating the claims definitions using electronic medical records [47].
Hsu et al. (2016) [48]	Taiwan National Health Research Insurance database from 1995 to 2010	AMI, stroke	AMI: 410.x; stroke: 433.x, 434.x, 436.x (ICD-9). Primary position.	Authors cite two studies that previously validated the accuracy of these ICD codes [49, 50].
Shih et al. (2016) [51]	Taiwan National Health Insurance Research Database from 1995 to 2012	AMI, ischemic stroke, in-hospital cardiovascular mortality	AMI: 410.x; ischemic stroke: 433.x, 434.x, 436.x (ICD-9); in-hospital cardiovascular mortality. Primary position.	Authors cited two studies for ischemic stroke validation only [50, 52].
Solomon et al. (2016) [53]	Medicare claims linked to Brigham and Women’s Hospital electronic medical record from 2006 to 2011	AMI, stroke, TIA	AMI: 410.x except 410.×2; stroke: 430.x, 431.x, 434.x, 436.x; TIA: 435.x (ICD-9). NR	Authors cited multiple studies demonstrating that using these specific ICD codes, there’s a 94% PPV for AMI, 96% PPV for stroke and 72–96% PPV for TIA [33, 54, 55].
Babazade et al. (2017) [56]	Inpatient databases from multiple U.S. states, HCUP database from 2009 to 2011	AMI, Acute coronary occlusion without AMI, postoperative pulmonary edema, cardiac arrest	AMI: 410; acute coronary occlusion without AMI: 411.81; pulmonary edema: 518.4; cardiac arrest: 997.1 (ICD-9). NR	NR
de Miguel-Yanes et al. (2017) [57]	Spanish National Hospital Discharge Database from 2002, 2006, 2010 and 2014	AMI, stroke, aortic aneurysm and dissection, acute limb ischemia	AMI: 410.xx; stroke: 431, 432.9, 433.× 1, 434.01, 434.11, 434.91; aortic aneurysm and dissection: 441.xx; acute limb ischemia: 440.21–440.24, 440.4, 444.22, 444.81, 445.02 (ICD-9). Primary position.	NR
Lindahl et al. (2017) [58]	SWEDEHEART from 2003 to 2013	AMI, ischemic stroke, HF, all cause death	AMI: I21, I22, I23; ischemic stroke: I63, I64; HF: I50, K761, I971, I110 (ICD-10). NR	NR
Policardo et al. (2017) [59]	Tuscany regional health system from 2008 to 2012	AMI, ischemic stroke, HF, lower extremity amputation	AMI: 410.xx; ischemic stroke: 430.xx, 431.xx, 432.xx, 434.xx, 436.xx; HF: 401.91, 402.01, 402.11, 402.91, 404.01, 404.3, 404.13, 404.93, 428.0, 428.1, 428.9; lower extremity amputation: 84.1x (ICD-9). NR	NR
Tung et al. (2017) [60]	Taiwan National Health Research Insurance database from 2009 to 2012	AMI, UA, stroke, resuscitation after cardiac arrest, all cause death, cardiac revascularization procedure	AMI: 410–410.9; UA: 411.1; stroke: 430–437; resuscitation after cardiac arrest: 427.5; revascularization: 36.0–36.03, 36.05–36.09, 36.1–36.99, V45.81 (ICD-9). NR	NR
Arinze et al. (2018) [61]	OptumLabs Data Warehouse from 2004 to 2014	AMI, stroke	AMI: 410; stroke: 434, 434.0, 434.00, 434.01, 434.1, 434.10, 434.11, 434.9, 434.90, 434.91 (ICD-9). NR	NR
Baviera et al. (2018) [62]	Administrative health databases of Lombardy from 2002 to 2014	ACS, stroke, all cause death, major amputation	ACS: 410.xx, 411; stroke: 430, 431, 434.xx, 436, 433.11; major amputation: 84.10, 84.13, 84.14, 84.15, 84.16, 84.17, 84.18, 84.19 (ICD-9). NR	Authors state that diagnoses were retrieved from hospital discharge charts, which have been validated across all Italian hospitals for reimbursement purposes.
Chan et al. (2018) [63]	Taiwan National Health Research Insurance database from 2000 to 2011	ACS, stroke, all cause death	ACS: 410; stroke: 430–438 (ICD-9). NR	NR
Wu et al. (2018) [64]	Taiwan National Health Research Insurance database from 2001 to 2013	AMI, stroke, HF, all cause death	AMI: 410.xx; stroke: 430.xx-437.xx; HF: 428.xx (ICD-9). NR	Authors cite an 88% PPV for AMI [65]. Authors do not report validation data specifically for stroke or HF.
Degli Esposti et al. (2018) [66]	Health-Assisted Subjects’ Database, Outpatients and Inpatients Pharmaceutical Drugs Database, Hospital Discharge Database from 2010 to 2011 (administrative databases of six Italian local health units)	AMI, other IHD, angina, cerebral artery occlusion, TIA, acute but ill-defined cerebrovascular disease	AMI: 410; IHD: 411; angina: 413; cerebral artery occlusion: 433, 434; TIA: 435; cerebrovascular disease: 436 (ICD-9). NR	NR
Hussain et al. (2018) [67]	Ontario Drug Benefit program database from 2002 to 2015	AMI, stroke, all cause death	AMI: I21.x, I22.x; stroke: I60.x I61.x I62.x I63.x, I64.x, H34.1, excluding I63.6 (ICD-10) NR	Authors cite studies validating ICD codes for AMI [68] and stroke [69].
Jin et al. (2018) [70]	U.S. Nationwide Readmissions Database from 2013	AMI, HF, cardiac revascularization procedure, aortic valve repair, cardiac ablation, cardioversion, cardiac catheterization, left atrial appendage closure, Implanted cardioverter defibrillator placement, pacemaker placement, ischemic stroke, hemorrhagic stroke, iatrogenic stroke	AMI: 410.x; HF: 428.x, 491.8, 491.9, 492.0, 492.6, 496.0; revascularization: 0.66, 36.1x, 36.2x; aortic valve repair: 35.00x, 35.01x, 35.1x, 35.2x, 35.3x, 35.05, 35.06; Cardiac ablation: 37.33, 37.34; Cardioversion 99.61, 99.62, 99.69; Cardiac catheterization: 37.21, 37.22, 37.23; Left atrial appendage closure 379, 373.6; Implanted cardioverter defibrillator placement: 37.94, 37.95, 37.96, 37.97, 37.98; pacemaker placement: 0.50, 0.51, 0.52, 0.53, 0.54, 37.7, 37.71, 37.72, 37.73, 37.74, 37.76, 37.80, 37.81, 37.82, 37.83, 37.85, 37.86, 37.87; Ischemic stroke 433.× 1, 434.× 1, 436; Hemorrhagic stroke 430, 431; Iatrogenic stroke 997.02 (ICD-9). Primary position.	Authors cite multiple studies that have demonstrated a PPV of 89–96% using these ICD codes [55, 71, 72].
Kim et al. (2018) [73]	Korean National Health Insurance Service from 2005 to 2015	AMI, ischemic stroke, hemorrhagic stroke, HF	AMI: I21, I22; ischemic stroke: I63, I64; hemorrhagic stroke: I60-I62; HF: I11.0, I50, I97.1 (ICD-10). NR	NR
Kiss et al. (2018) [74]	National Health Insurance Fund database 2010–2013	AMI, stroke	AMI: I21–24; stroke: I61–63 (ICD-10). NR	NR
Ohm et al. (2018) [75]	Swedish national quality registry SWEDEHEART, sub-registry SEPHIA from 2006 to 2013	AMI, ischemic stroke, CV death	AMI: I21.0–4, I21.9, I22.0–1, I22.8–9; ischemic stroke: I63.0–9 (ICD-10); and CV death: I46.1, I46.9. NR	NR
Winell et al. (2018) [76]	National Hospital Discharge Register, National Causes of Death Register from 2000 to 2011	ACS, ischemic stroke	ACS: 410, 411 (ICD-9) I20.0, I21, I22 (ICD-10); stroke: 433–436, excluding 434.9x (ICD-9), I63, I64, G45 (ICD-10). NR	Authors cite multiple studies which have validated the use of these ICD codes for this database [77, 78].
Yang et al. (2018) [79]	Taiwan National Health Research Insurance database from 1996 to 2013	AMI, ischemic stroke	AMI: 410; ischemic stroke: 433–436 (ICD-9). NR	NR
Young et al. (2018) [80]	Optum™ Integrated Real World Evidence Electronic Health Records and Claims de-identified database from 2006 to 2016	AMI, stroke, CV death	AMI: 410.xx (ICD-9), I21.xx, I22.xx (ICD-10); stroke: 430.xx, 431.xx, 434.xx, 436.xx (ICD-9), I60.xx, I61.xx, I63.3-I63.9, I66.xx (ICD-10); CV death (defined as death within 30 days of AMI, stroke or the following ICD codes): 411.xx-414.xx, 415.xx-417.xx, 420.xx-427.xx, 428.xx, 429.xx, 432.xx, 433.xx, 435.xx, 437.xx, 785.51 (ICD-9), I20.x, I23.xx-I25.xx, I46.9, I30.xx-I52.xx, I62.xx, I63.0-I63.2, I64.xx, I65.xx, I67.xx, I68.xx, R57.0 (ICD-10). NR	NR
Arnaout et al. (2019) [81]	California Healthcare Cost and Utilization Project database from 2005 to 2009	AMI, stroke, HF	AMI: 410.00–410.92; stroke: 434.91, 434.11, 459.0; HF: 402.01, 402.11, 402.91, 404.91, 404,93, 425.0–425.1, 425.11, 425.18, 425.2, 425.3, 425.4, 425,5, 425.7–425.9, 428,0–428.2, 428.20–428.21, 428.22–428.23, 428.30–428.33, 428.4, 428.40–428.43, 428.9 (ICD-9). NR	Authors cite multiple studies that have demonstrated good sensitivity and specificity for AMI, stroke and HF using these ICD codes [10–12].
Chen et al. (2019) [82]	Taiwan National Health Research Insurance database from 2000 to 2008	IHD, HF	IHD: 410–414; HF: 428 (ICD-9). NR	Authors cite study that validated ICD codes for AMI [65].
Giral et al. (2019) [83]	Système National d’Information Inter-Régimes de l’Assurance Maladie from 2012 to 2014	Coronary event, cerebrovascular event, other vascular event	Coronary event: I20, I21, I22, I23, I24; cerebrovascular event: G45, G46, H34.0, H34.1, H34.2, H34.9, I60, I61, I62, I63, I64, I65, I66, I67.0; other vascular event: I70.01, I70.21, I70.81, I70.91, I71.0, I71.1, I71.3, I71.5, I71.8, I72, I74, K55.9 (ICD-10). NR	NR
Hsueh et al. (2019) [84]	Taiwan National Health Research Insurance database from 2000 to 2013	IHD, HF	IHD: 410–414; HF: 428 (ICD-9). NR	Authors cite study that validated ICD codes for AMI using this database [65].
Kim et al. (2019) [85]	South Korean National Health Information Database from 2006 to 2014	IHD, ischemic stroke, cardiac revascularization procedure	IHD: I21–24; ischemic stroke: I63, I65, I66; (ICD-10). Authors did not cite ICD codes for cardiac revascularization procedures. Primary position.	NR
Kuo et al. (2019) [86]	Taiwan National Health Research Insurance database from 1999 to 2010	AMI, IHD, HF, cerebrovascular disease, all cause death	AMI: 410, 412; IHD: 411, 413, 414, V45.81, V45.82, 00.66C, 36.0, 36.1, 36.2, 36.3, 36.9, 88.5, 37.8; HF: 428; cerebrovascular disease: 430–437, 438, V12.54, 38.11, 38.12 (ICD-9) NR	Authors cited multiple studies that previously validated the accuracy of these ICD codes [50, 65, 87–90].
Li et al. (2019) [91]	Taiwan National Health Insurance Research Database from 2000 to 2011	CAD, HF, atrial fibrillation, stroke	CAD: 410–414; AMI: 410; HF: 428; atrial fibrillation: 427.31; stroke: 430–438 (ICD-9). NR	NR
Mureddu et al. (2019) [92]	Italian National Registry of Hospital Discharge Records (ItHDR) from 2004 to 2008, 2009–2010, 2011–2015	AMI, stroke, all-cause death	AMI: 410, 412; stroke: 433.×1, 434.× 1 (with the exclusion of 431, 432.x) (ICD-9). NR	NR
Lin et al. (2019) [93]	Taiwan National Health Insurance Research Database from 1999 to 2011	AMI, ischemic stroke	AMI: 410.x; ischemic stroke: 433.x, 434.x, 436 (ICD-9). NR	Authors cite two studies validating ICD codes for AMI and stroke using this database [50, 65].
Bourrier et al. (2020) [94]	The Manitoba Health Insurance Registry, Medical Services, Discharge Abstract Database, and Diagnostic Services of Manitoba (Manitoba, Canada) from 2007 to 2014	AMI, stroke, HF	AMI: 410, 411, I21, I22, I23; stroke: 431, 432, 434, I61, I62, I63, I64; HF: 428, I50 (ICD-9 and 10). NR	NR
Lin et al. (2020) [95]	Taiwan National Health Insurance Research Database from 2008 to 2012	AMI, stroke, PCI, CABG, all-cause mortality.	AMI: 410–410.9, stroke: 430–437, PCI: 36.0–36.03, 36.05–36.09; CABG: 36.1–36.99, V45.81 (ICD-9). NR	NR
Madsen et al. (2020) [96]	Copenhagen General Population Study from 2003 to 2015	AMI, CABG, PCI, stroke, CV death.	AMI: I21-I22; CABG: NOMESCO: KNFA-KFNE; PCI: NOMESCO: KFNG00–05); stroke: I60-I61, I63-I64; CV death: I01-I87, I95-I99, R96 (ICD-10) NR	NR
Petersen et al. (2020) [97]	Danish health data including the clinical laboratory information system (LABKA), the Danish National Patient Registry, the Aarhus University Prescription Database (AUPD) and the Danish Civil Registration System from 2006 to 2014.	AMI, stroke, all-cause mortality.	AMI: I21; stroke: I61, I62, I64, I65 (ICD-10). NR	NR
Seong et al. (2020) [98]	National Health Insurance Service-Elderly Cohort Database (NHIS-ECD) by the Korean National Health Insurance Service (KNHIS) from 2003 to 2008.	AMI, IHD, stroke, and CV death.	AMI: I21-I23, I25.2; IHD: I20-I25; stroke: I63; CV death: I60-I69 (ICD-10). Any position.	NR

*Abbreviations*: *NR* Not reported, *ICD* International Classification of Diseases, *CV* Cardiovascular, *AMI* Acute myocardial infarction, *IHD* Ischemic heart disease, *ACS* Acute coronary syndrome, *CAD* Coronary artery disease, *UA* Unstable angina, *PVD* Peripheral vascular disease, *HF* Heart failure, *TIA* Transient ischemic attack, *PCI* Percutaneous coronary intervention, *CABG* Coronary artery bypass graft

**Table 2:** Most common components of MACE (*N* = 58 studies)

MACE Components	ICD-9	ICD-10	Both ICD-9/10
**Individual components**			
AMI (*n* = 45)	29	14	2
Acute coronary syndrome / Ischemic Heart disease (*n* = 18)	12	5	1
Stroke (*n* = 50)	30	17	3
Heart failure (*n* = 15)	12	2	1
All-cause death (*n* = 15)	10	5	0
CV death (*n* = 9)	1	6	2
Revascularization (*n* = 11)	7	4	0
Other (*n* = 19)	15	3	1
**Combinations**			
Three-point MACE: AMI + Stroke+CV death (*n* = 5)	1	3	1
Four-point MACE: AMI + Stroke+UA + CV death (*n* = 0)	0	0	0
Five-point MACE: AMI + Stroke+UA + HF + CV death (*n* = 0)	0	0	0
AMI + stroke+all-cause death (*n* = 8)	5	3	0
AMI + stroke+HF + CV death (*n* = 0)	0	0	0
AMI + stroke+HF + all-cause death (*n* = 2)	1	1	0

*Abbreviations*: *MACE* Major adverse cardiovascular events, *ICD* International Classification of Diseases, *AMI* Acute myocardial infarction, *CV* Cardiovascular, *UA* Unstable angina, *HF* Heart failure

**Table 3:** Most commonly used MACE components

MACE Components	Frequency, n (%)
AMI, stroke	9 (15.5)
AMI, stroke, all-cause death	8 (13.8)
AMI, stroke, CV death	5 (8.6)
ACS/IHD, stroke	3 (5.2)
AMI, stroke, HF	3 (5.2)
Other definitions (≤2 studies using each)	30 (51.7)

*Abbreviations:* *MACE* Major adverse cardiovascular events, *AMI* Acute myocardial infarction, *CV* Cardiovascular, *ACS* Acute coronary syndrome, *IHD* Ischemic heart disease, *HF* Heart failure

### MACE component definitions

#### Acute myocardial infarction

There were 45 studies that included AMI as a component of MACE (Table 2). Of these, 64.4% (29/45) defined outcomes using ICD-9-CM and 31.1% (14/45) used ICD-10-CM. Among these studies, 20% (9/45) defined AMI in the primary diagnosis position, 6.7% (3/45) in any position, and 73.3% (33/45) did not report the position used. The most common diagnosis codes were: 410.xx, making up 72% (21/29) of the ICD-9-CM studies; and I21.xx, I22.xx, making up 35.7% (5/14) of the ICD-10-CM studies (Table 4).

**Table 4:** ICD codes by clinical outcome

Outcome	ICD-9 code	ICD-10 Code	Both ICD-9/10 Codes
AMI (*n* = 45)	1. 410.xx (*n* = 21) 2. 410.xx, 412.xx (*n* = 3) 3. Other (*n* = 5)	1. I21.xx, I22.xx (*n* = 5) 2. I21.xx (*n* = 4) 3. Other (*n* = 5)	1. 410.xx, I21.xx, I22.xx (*n* = 1) 2. 410–411.xx, I21-I23.xx (*n* = 1)
ACS/IHD (*n* = 18)	1. 410.xx-414.xx (*n* = 3) 2. 410.xx, 411.xx (*n* = 2) 3. Other (*n* = 7)	1. I21.xx-I24.xx (*n* = 2) 2. Other (*n* = 3)	1. 410.xx, 411.xx, I20.0, I21.xx, I22.xx (*n* = 1)
Stroke (*n* = 50)	1. 433.xx, 434.xx, 436.xx (*n* = 4) 2. 430.xx-437.xx (*n* = 4) 3. Other (*n* = 22)	1. I63.xx, I65.xx, I66.xx (*n* = 3) 2. I63.xx (*n* = 3) 3. Other (*n* = 11)	2. 430.xx, 431.xx, 434.xx, 436.xx, I60.xx, I61.xx, I63.3-I63.9, I66.xx (*n* = 1) 3. 433.xx-436, excluding 434.9x, I63, I64, G45 (*n* = 1) 4. 431.xx-432.xx, 434.xx, I61.xx-I64.xx (*n* = 1)
Heart Failure (*n* = 15)	1. 428.xx (*n* = 6) 2. Other (*n* = 6)	1. I11.0, I50.xx, I97.1 (*n* = 1) 2. I50.xx, K76.1, I97.1, I11.0 (*n* = 1)	1. 428.xx, I50.xx (*n* = 1)
CV Death (*n* = 9)	1. None listed (*n* = 1)	1. I00.xx-I99.xx (*n* = 2) 2. I46.1, I46.9 (*n* = 1) 3. Other or none listed (*n* = 3)	1. 411.xx-414.xx, 415.xx-417.xx, 420.xx-427.xx, 428.xx, 429.xx, 432.xx, 433.xx, 435.xx, 437.xx, 785.51, I20.xx, I23.xx-I25.xx, I46.9, I30.xx-I52.xx, I62.xx, I63.0-I63.2, I64.xx, I65.xx, I67.xx, I68.xx, R57.0 (*n* = 1) 2. 410.xx-414.xx, 428.xx, 430.xx-438.xx, I20.xx–I25.xx, I50.xx, I60.xx-I69.xx (*n* = 1)

*Abbreviations*: *ICD* International Classification of Diseases, *AMI* Acute myocardial infarction, *ACS* Acute Coronary Syndrome, *IHD* Ischemic Heart Disease, *CV* Cardiovascular

#### Acute coronary syndrome / ischemic heart disease

There were 18 studies that defined ACS/IHD as a component of MACE. ICD-9-CM diagnosis codes only were used in 66.7% (12/18) of studies and 27.7% (5/18) used ICD-10-CM only. Studies used the primary diagnosis position 22.2% (4/18), any position 11.1% (2/18), and did not report the position 66.7% (12/18) of the time. The most common diagnosis codes were: 410–414.xx, 25% (3/12) of the ICD-9-CM studies; and I21-I24.xx, 40% (2/5) of the ICD-10-CM studies.

#### Stroke

There were 50 studies that included stroke, either ischemic or hemorrhagic, as a component of MACE. Of these, 60% (30/50) utilized ICD-9-CM only and 34% (17/50) used ICD-10-CM only. The primary diagnosis position was used 24% (12/50) of the time, any position used 8% (4/50), and no position reported 68% (34/50) of the time. Diagnosis codes used to define stroke were highly variable with no clear most common definition. Codes included: 433.xx, 434.xx, 436.xx, 13.3% (4/30) of ICD-9-CM studies; 430–437.xx, 13.3% (4/30) of ICD-9-CM studies; I63.xx, I65.xx, I66.xx, 17.6% of ICD-10-CM studies (3/17); and I63.xx, 17.6% (3/17) of ICD-10-CM studies.

#### Heart failure

A total of 15 studies included HF as a component of MACE. Of these studies, 80% (12/15) used ICD-9-CM only, and 13.3% (2/15) used ICD-10-CM only. The primary diagnosis position was used 20% (3/15) of the time but was otherwise not reported in 80% (12/15) of studies. The most common diagnosis codes used were: 428.xx, 50% (6/12) of ICD-9-CM studies; I11.0, I50.xx, I97.1, 50% (1/2) of ICD-10-CM studies; and I50.xx, K76.1, I97.1, I11.0, 50% (1/2) of ICD-10-CM studies.

#### All-cause death and cardiovascular death

There were nine studies that included cardiovascular death as a component of MACE. Of these, 11.1% (1/9) of studies utilized ICD-9-CM codes only for MACE components and 66.7% (6/9) used ICD-10-CM. The diagnosis position for cardiovascular death was primary 11.1% (1/9), any position 33.3% (3/9), and not reported 55.6% (5/9) of the time. The most common diagnosis codes used were: No codes listed, 100% (1/1) for studies using ICD-9-CM only; and I00.xx-I99.xx, 33.3% (2/6) for those using ICD-10-CM only. There were 15 studies including all-cause death as a component of MACE. Due to the nature of all-cause death, diagnosis codes positions are not necessary and were not reported.

## Discussion

In our systematic review, we found substantial heterogeneity for MACE composite endpoints used in the literature. The two most common composite MACE definitions were “AMI and stroke”, and “AMI, stroke, and all-cause death,” but they only made up 15.5 and 13.8% of studies, respectively. Compared to MACE definitions used in RCTs, only 8.6% of included observational studies had definitions aligned with three-point MACE and none match four-point or five-point MACE. A large majority of studies included AMI and stroke but there was a lack of consensus with other components included in composite MACE endpoints. For instance, over half of included publications defined MACE using a definition that only was concordant with up to one other study. This diversity makes it challenging to compare findings across studies or to aggregate multiple study results for meta-analyses or systematic reviews when considering different treatment or research questions. Addressing the heterogeneity of MACE definitions used in practice requires attention due to the advantages of the MACE endpoint. Utilizing MACE as a composite outcome can potentially reduce the number of patients that need to be enrolled or identified in a retrospective cohort study, and reduce the follow-up time necessary to observe differences between different treatment groups [99]. These benefits not only potentially reduce research costs but can more expediently answer clinical questions, leading to improved patient care. Therefore, given that MACE endpoints will likely see increasing use as time goes on, there should be a continued effort to standardize and transparently report MACE definitions used in observational studies.

In 2007, findings similar to our study were observed when comparing prospective trials conducted for percutaneous coronary interventions [100]. However, although the FDA in 2008 and the EMA in 2012 attempted to standardize the MACE endpoint definition for RCTs, we found a continued discordance between how observational studies define MACE. Furthermore, we found that the majority of studies did not include mention of the diagnosis position used to define outcomes. Lack of this information prevents the ability to distinguish between incident and prevalent outcomes. While the primary position has been historically used in observational studies to define incident outcomes, the use of only primary diagnosis positions, compared to using secondary positions, may underestimate the rates of MACE for prevalent conditions [101]. The lack of reporting of diagnosis position prevents the ability to make this distinction and prohibits the full interpretation of findings.

Heterogeneity not only existed in defining MACE components but also in which ICD-9-CM and ICD-10-CM codes were used to define each individual MACE component. In our study, none of the top five components of MACE had a consensus ICD-9-CM or ICD-10-CM code that was used among all of the studies. These discrepancies contribute additional variability to our findings, as outcome classification, assuming a standard definition, inherently relies on the providers and medical billers accurately coding diseases. For example, in a study conducted in 11 Canadian emergency departments, the authors reported about 82–86% of agreement with regards to coding hospital conditions [102]. Additionally, a study looking at stroke data collected through the United States Paul Coverdell National Acute Stroke Program found that discordance existed with how strokes were coded, especially at smaller hospitals (< 200 beds) and when differentiating between ischemic strokes, transient ischemic attacks, and subarachnoid and intracerebral hemorrhages [103].

There have been many attempts to validate diagnosis or procedure codes for various MACE component outcomes [32, 104]. Research has predominantly focused on the older ICD-9-CM codes, particularly studies using U.S. administrative data, but studies utilizing ICD-10-CM have more recently been published. Using data from the Centers for Medicare and Medicaid Services, a 2018 study found that the most accurate ICD-9-CM code for coding AMI was 410.xx, with a positive predictive value of 67% [104]. Additionally, the U.S. FDA commissioned the Mini-Sentinel pilot program to systematically assess the validation of common healthcare outcomes in administrative databases [32]. These studies were important steps to identify standard outcome definitions for use across disparate data sources and populations. As part of the program, systematic literature reviews from the U.S. and Canada found that the most accurate ICD-9-CM code for heart failure was 428.xx, with a positive predictive value of 84–100%, while the most accurate ICD-9-CM codes for stroke were 430.x, 431.x and 434.x, all with separate positive predictive values > 80%, and 436.x, which had a positive predictive value > 70% [32, 33]. The Mini-Sentinel program also assessed the ICD-9-CM codes, 410.× 0 or 410.× 1, for AMI and found a positive predictive value of 76.3–94.3% [105]. Fewer validations exist for ICD-10-CM codes. One study conducted in Japan attempted to validate the ICD-10-CM code I21.x for AMI and found a positive predictive value of 82.5% [106]. Another study in Canada found the positive predictive value for correctly coding stroke was 92% for the ICD-10-CM codes, I60.x-I61.x, I63.x-I64.x, H34.1 and G45.x [69]. Based on our findings, the most common ICD-9-CM and 10-CM codes used for AMI were also the most validated, 410.xx and I21.xx, respectively. The most validated and most common ICD-9-CM code used for heart failure was 428.xx. On the other hand, stroke had some discrepancies when comparing the most validated versus the most commonly used ICD codes. This discordance likely existed because of the differences with which stroke can be defined, particularly whether to include acute ischemic strokes with transient ischemic attacks, intracerebral hemorrhages or subarachnoid hemorrhages [69].

There are several limitations to our study. We excluded papers published before 2010, prior to the first published reviews of the Mini-Sentinel program, in order to present a contemporary review of the subject [32]. However, this cutoff likely skewed our results towards more modern MACE definitions and excluded different definitions that were potentially used for older publications. Older studies likely exhibited greater variety in the outcome definitions used. Additionally, because the words “major adverse cardiovascular events” were rarely explicitly written, we had to include any composite definitions that were thought to represent MACE but were listed using another composite name, such “acute cardiovascular events,” based on the discretion of the reviewer. This method potentially excluded studies that used variations of MACE endpoints, but did not conform to similar naming conventions.

## Conclusion

Significant heterogeneity exists in how MACE is defined and which ICD-9-CM and 10-CM codes are used to represent each respective MACE outcome in observational studies using administrative databases. The utility of future studies will improve with the use of validated definitions of AMI, stroke, cardiovascular mortality, unstable angina, and HF for the evaluation of MACE outcomes. Further, investigators should ensure that both the ICD diagnosis codes and the code positions are reported in a transparent way. Given the significant heterogeneity that already exists across administrative databases, we recommend the use of more standardized MACE definitions and corresponding ICD-9-CM and ICD-10-CM codes. This practice will allow researchers to more accurately compare findings across studies improve the reproducibility of observational studies, and decrease the potential for misleading conclusions.

## Supplementary Information

**Additional file 1: Supplementary Text 1.** Systematic Review Protocol.

**Additional file 2: Supplementary Table 1.** PRISMA 2020 Checklist.

**Additional file 3: Supplementary Text 2.** Search terms.

## Data Availability

Data sharing is not applicable to this article as no datasets were generated or analyzed during the current study.

## Abbreviations

ACS: Acute coronary syndrome
ACS/IHD: Acute coronary syndrome or ischemic heart disease
AMI: Acute myocardial infarction
CABG: Coronary artery bypass graft
CAD: Coronary artery disease
CV: Cardiovascular
HF: Heart failure
ICD: International Classification of Diseases
ICD-9-CM: International Classification of Diseases, 9th Revision, Clinical Modification
ICD-10-CM: International Classification of Diseases, 10th Revision, Clinical Modification
IHD: Ischemic heart disease
MACE: Major adverse cardiovascular events
PCI: Percutaneous coronary intervention
PRISMA: Preferred Reporting Items for Systematic Reviews and Meta-Analysis
PRISMA-P: Preferred Reporting Items for Systematic Reviews and Meta-Analysis Protocols
PROSPERO: International Prospective Register of Systematic Reviews
PVD: Peripheral vascular disease
RCT: Randomized controlled trial
TIA: Transient ischemic attack
UA: Unstable angina
