# Supplementary material for: Major adverse cardiovascular event definitions used in observational analysis of administrative databases: a systematic review
Source: BMC Med Res Methodol. 2021 Nov 6;21:241. doi: 10.1186/s12874-021-01440-5 (PMC8571870; doi:10.1186/s12874-021-01440-5)
Supplement: Supplementary file 1 — Additional file 1: Supplementary Text 1. Systematic Review Protocol. [file 12874_2021_1440_MOESM1_ESM.docx]

**Supplementary Text 1. Systematic Review Protocol**

**Protocol: Major Adverse Cardiovascular Event Definitions Used in Observational Analysis of Administrative Databases: A Systematic Review**

Authors: Elliott Bosco, PharmD, PhD,^1,2*^ Leon Hsueh, MD,^3*^ Kevin W. McConeghy, PharmD, MS,^1,2,4^ Stefan Gravenstein, MD, MPH,^1,2,3,4^ Elie Saade, MD^5,6^

Affiliations:

^1^Department of Health Services, Policy, and Practice, Brown University School of Public Health, Providence, Rhode Island, USA;

^2^Center for Gerontology and Healthcare Research, School of Public Health, Brown University School of Public Health, Providence, Rhode Island, USA;

^3^Department of Medicine, Warren Alpert Medical School of Brown University, Providence, Rhode Island, USA;

^4^Center of Innovation in Long-Term Services and Supports, Providence Veterans Affairs Medical Center, Providence, Rhode Island, USA;

^5^Division of Infectious Diseases and HIV Medicine, University Hospitals Cleveland Medical Center, Cleveland, OH, USA;

^6^School of Medicine, Case Western Reserve University, Cleveland, OH, USA.

Sponsor/Funding: Unfunded.

Contributions to Protocol: All authors contributed to development of the systematic review protocol and the guarantor is Elliott Bosco, PharmD, PhD.

**Corresponding Author Address:** Department of Health Services, Policy, and Practice, Brown University School of Public Health, 121 South Main Street, Box G-S121-3, Providence, RI, 02912

**PROSPERO Registration:** Not registered.

**Rationale:** Cardiovascular disease is the leading cause of death in the United States, making it a common target for interventional research [1, 2]. Due to this, the composite endpoint of “major adverse cardiovascular events” (MACE) is an increasingly common primary outcome of interest. While MACE is now a better-defined and more ubiquitous outcome among RCTs, its use in observational studies to assess the safety and real-world effectiveness of therapies remains less clear. Observational studies using large administrative databases can evaluate population-level use and outcomes of therapies in an efficient and cost-effective way [5]. To allow for useful comparisons between observational studies and RCTs, improved standardization and transparency is needed [7, 8]. For these reasons, the current use of MACE in observational studies warrants further investigation.

**Objectives:** The study objectives were:

1. Assess each study’s definition of MACE components (e.g., AMI, stroke).
2. Assess the diagnostic criteria used for outcome ascertainment such as codes used and position of codes.
3. Assess whether outcomes had been validated.

**Eligibility Criteria:**

**Inclusion criteria:**

- Study conducted using claims, electronic health records, a registry, or real-world database.
- MACE composite outcome used as primary or secondary study outcome. Note: Full-text review only, not required for abstract screening. Only MACE components (e.g., AMI, stroke, HF) were required to be mentioned in abstracts included during screening.
- MACE outcomes defined using ICD-9-CM and ICD-10-CM codes.

**Exclusion criteria:**

- Non-English
- Unable to access
- Study is abstract only, systematic review, case report, commentary or editorial.
- ICD-9-CM and ICD-10-CM codes not provided

Note, during abstract screening: Retain abstract during screening if there is uncertainty about any of the factors above. Articles will be assessed for inclusion and exclusion criteria based on full-text review.

**Information Sources and Search Strategy:**

Searches will be conducted in Medline and EMBASE databases. The final searches will consider studies published between January 1, 2010 to the date of the search (2020) to include contemporary articles published in the last 10 years. The following searches will be used.

Medline:

((("cardiovascular events"[All Fields] OR "cardiovascular event"[All Fields] OR "cardiac events"[All Fields] OR "cardiac event"[All Fields]) AND ("prophylax*"[Title/Abstract] OR "prevent*"[Title/Abstract]) AND ("Database Management Systems"[MeSH Terms] OR "medical records systems, computerized"[MeSH Terms] OR "databases, factual"[MeSH Terms] OR "Databases as topic"[MeSH Terms] OR "Medical Record Linkage"[MeSH Terms] OR "Incidence"[MeSH Terms] OR "International Classification of Diseases"[MeSH Terms] OR "Current procedural terminology"[MeSH Terms] OR "Electronic health records"[MeSH Terms] OR "Insurance claim review"[MeSH Terms] OR "Outcome assessment"[All Fields] OR "Data Warehouse"[All Fields] OR "ICD-9"[All Fields] OR "ICD-10"[All Fields] OR "CPT"[All Fields] OR "Current procedural terminology"[All Fields] OR "database"[All Fields] OR "databases"[All Fields] OR "population surveillance"[All Fields] OR "data collection"[All Fields] OR "automatic data processing"[All Fields] OR "patient record"[All Fields] OR "patient discharge"[All Fields] OR "hospital records"[All Fields] OR ("claims"[Text Word] AND "administrative"[Text Word]) OR ("data"[Text Word] AND "administrative"[Text Word]) OR "international statistical classification"[All Fields] OR "International Classification of Diseases"[All Fields] OR "population surveillance"[All Fields] OR "hospital discharge data"[All Fields] OR ("health"[Text Word] AND "administrative"[Text Word]) OR ("electronic"[Text Word] AND "record"[Text Word]) OR ("medical"[Text Word] AND "record"[Text Word]) OR ("health"[Text Word] AND "record"[Text Word]))) NOT ("Editorial"[Publication Type] OR "Meta-Analysis"[Publication Type] OR "Comment"[Publication Type] OR "case reports"[Publication Type] OR "Review"[Publication Type])) AND ("humans"[MeSH Terms] AND "English"[Language] AND "adult"[MeSH Terms])

EMBASE:

('factual database'/exp OR 'factual database*' OR 'medical record'/exp OR (health NEAR/2 administrative) OR (administrative NEAR/2 data) OR (electronic NEAR/2 record*) OR (health NEAR/2 record*) OR (hospital NEAR/2 record*) OR 'international classification of diseases'/exp OR 'international classification of disease*' OR 'icd-9' OR 'icd-9-cm' OR 'icd-10' OR 'icd-10-cm' OR 'current procedural terminology'/exp OR 'current procedural terminology' OR (('insurance'/exp OR 'insurance') AND ('database'/exp OR 'database' OR database*)) OR 'claim*') AND ('cardiovascular event':ab,ti OR 'cardiovascular events':ab,ti OR 'cardiac event':ab,ti OR 'cardiac events':ab,ti OR 'acute cardiovascular event':ab,ti OR 'acute cardiovascular events':ab,ti OR 'acute cardiac event':ab,ti OR 'acute cardiac events':ab,ti OR 'adverse cardiovascular event':ab,ti OR 'adverse cardiovascular events':ab,ti OR 'adverse cardiac event':ab,ti OR 'adverse cardiac events':ab,ti) NOT ('editorial'/it OR 'meta analysis'/it OR 'case report'/it OR 'letter'/it OR 'chapter'/it) AND [humans]/lim AND [clinical study]/lim AND [english]/lim AND [adult]/lim AND [embase]/lim NOT ([embase]/lim AND [medline]/lim)

**Data Management, Abstract Screening, and Full-text Review:**

Study records from all searches will be imported into EndNote and duplicates will be removed. Study records will be loaded into Abstrackr, the semi-automated abstract screening tool (<https://www.brown.edu/public-health/cesh/resources/software>). After abstract screening, full-text articles will be obtained.

We will extract data from the final included articles using a Excel-based data extraction form. The entire process will be piloted with 20 articles by three reviewers and modified based on the author’s findings. Any disagreements will be discussed among the reviewers until a consensus is reached. If a disagreement persists another author will be consulted for resolution.

**Data Elements Extracted:**

Elements extracted will include:

- Year
- Authors
- Study title
- Database used in study
- The components of MACE composite endpoints (e.g., AMI, stroke, HF)
- The ICD-9-CM and ICD-10-CM codes used to define MACE composite endpoints.
- The position of the ICD-9-CM and ICD-10-CM codes used to define the endpoints.
- Whether a validation of the outcome definition was performed in the study or if a prior validation study had been performed.

**Pilot Phase:**

Three reviewers will complete abstract screening, full-text review, and extraction of data for the same 20 studies and will then confer to discuss necessary changes to the study process.

**Protocol Modifications After Pilot Phase:**

The original protocol was modified after piloting so that MACE did not need to be explicitly mentioned in the abstract and we instead accepted mention of MACE components (e.g., AMI, stroke, HF, etc) during abstract screening. The inclusion of a MACE component endpoint was then verified on full-text review. This change to the protocol was made as abstract screeners noticed that a MACE composite endpoint was variably mentioned in the abstract, but was commonly included in the full-text. Individual MACE components were almost always reported in the abstract, even when the composite MACE endpoint was not mentioned.
