# Supplementary material for: Major adverse cardiovascular event definitions used in observational analysis of administrative databases: a systematic review
Source: BMC Med Res Methodol. 2021 Nov 6;21:241. doi: 10.1186/s12874-021-01440-5 (PMC8571870; doi:10.1186/s12874-021-01440-5)
Supplement: Supplementary file 3 — Additional file 3: Supplementary Text 2. Search terms. [file 12874_2021_1440_MOESM3_ESM.docx]

**Supplementary Text 2. Search terms**

**PubMed Search Strategy:**

((("cardiovascular events"[All Fields] OR "cardiovascular event"[All Fields] OR "cardiac events"[All Fields] OR "cardiac event"[All Fields]) AND ("prophylax*"[Title/Abstract] OR "prevent*"[Title/Abstract]) AND ("Database Management Systems"[MeSH Terms] OR "medical records systems, computerized"[MeSH Terms] OR "databases, factual"[MeSH Terms] OR "Databases as topic"[MeSH Terms] OR "Medical Record Linkage"[MeSH Terms] OR "Incidence"[MeSH Terms] OR "International Classification of Diseases"[MeSH Terms] OR "Current procedural terminology"[MeSH Terms] OR "Electronic health records"[MeSH Terms] OR "Insurance claim review"[MeSH Terms] OR "Outcome assessment"[All Fields] OR "Data Warehouse"[All Fields] OR "ICD-9"[All Fields] OR "ICD-10"[All Fields] OR "CPT"[All Fields] OR "Current procedural terminology"[All Fields] OR "database"[All Fields] OR "databases"[All Fields] OR "population surveillance"[All Fields] OR "data collection"[All Fields] OR "automatic data processing"[All Fields] OR "patient record"[All Fields] OR "patient discharge"[All Fields] OR "hospital records"[All Fields] OR ("claims"[Text Word] AND "administrative"[Text Word]) OR ("data"[Text Word] AND "administrative"[Text Word]) OR "international statistical classification"[All Fields] OR "International Classification of Diseases"[All Fields] OR "population surveillance"[All Fields] OR "hospital discharge data"[All Fields] OR ("health"[Text Word] AND "administrative"[Text Word]) OR ("electronic"[Text Word] AND "record"[Text Word]) OR ("medical"[Text Word] AND "record"[Text Word]) OR ("health"[Text Word] AND "record"[Text Word]))) NOT ("Editorial"[Publication Type] OR "Meta-Analysis"[Publication Type] OR "Comment"[Publication Type] OR "case reports"[Publication Type] OR "Review"[Publication Type])) AND ("humans"[MeSH Terms] AND "English"[Language] AND "adult"[MeSH Terms])

**EMBASE Search Strategy:**

('factual database'/exp OR 'factual database*' OR 'medical record'/exp OR (health NEAR/2 administrative) OR (administrative NEAR/2 data) OR (electronic NEAR/2 record*) OR (health NEAR/2 record*) OR (hospital NEAR/2 record*) OR 'international classification of diseases'/exp OR 'international classification of disease*' OR 'icd-9' OR 'icd-9-cm' OR 'icd-10' OR 'icd-10-cm' OR 'current procedural terminology'/exp OR 'current procedural terminology' OR (('insurance'/exp OR 'insurance') AND ('database'/exp OR 'database' OR database*)) OR 'claim*') AND ('cardiovascular event':ab,ti OR 'cardiovascular events':ab,ti OR 'cardiac event':ab,ti OR 'cardiac events':ab,ti OR 'acute cardiovascular event':ab,ti OR 'acute cardiovascular events':ab,ti OR 'acute cardiac event':ab,ti OR 'acute cardiac events':ab,ti OR 'adverse cardiovascular event':ab,ti OR 'adverse cardiovascular events':ab,ti OR 'adverse cardiac event':ab,ti OR 'adverse cardiac events':ab,ti) NOT ('editorial'/it OR 'meta analysis'/it OR 'case report'/it OR 'letter'/it OR 'chapter'/it) AND [humans]/lim AND [clinical study]/lim AND [english]/lim AND [adult]/lim AND [embase]/lim NOT ([embase]/lim AND [medline]/lim)
